# Cardiovascular-kidney-metabolic: Hype or a focus on front-line health?

**DOI:** 10.1515/jtim-2025-0049

**Published:** 2025-12-12

**Authors:** Xinyi Cai, Hao Huang, Tuo Li

**Affiliations:** Department of Endocrinology, Changzheng Hospital, Second Military Medical University, Shanghai, China

## Background

Cardiovascular-kidney-metabolic (CKM) syndrome, recently defined by the American Heart Association (AHA) in a 2023 presidential advisory,^[1]^ represents a global public health crisis with prevalence exceeding 30% in adults aged over 40. The AHA staging system (Stages 0–4) categorizes disease progression from adipose dysfunction to organ damage. However, it omits hepatic biomarkers, even though liver fat accumulation precedes insulin resistance by 5–7 years. Second, overemphasis on cardio-renal outcomes ignores pan-metabolic vasculopathy—systemic vascular injury affecting cerebral/ peripheral beds through shared mechanisms (*e.g*., fetuin-A mediated endothelial dysfunction). Third, unaddressed clinical gaps in early-diagnosis criteria, pharmacokinetic interactions between sodium-glucose cotransporter-2 inhibitor (SGLT2i) and glucagon-like peptide-1 receptor agonist (GLP-1RAs), and resource-appropriate screening. These limitations necessitate a liver-incorporated paradigm that preserves CKM’s clinical utility while enabling earlier intervention.

## Ectopic fat depots and organ metabolic disorders

CKM often originates from excessive or dysfunctional adipose tissue.^[1]^ While body mass index (BMI) and weight are commonly viewed as the measures of overweight and obesity, there are individuals who are under metabolic dysfunction but in normal weight, characterized with silent signs such as ectopic fat deposition and insulin resistance (IR).^[2]^ Consequently, we recommend that the accumulation of visceral fat in non-obese individuals should be taken as priority as well. Although visceral fat constitutes only a small fraction of total body fat, it is active and wellvascularized, capable of releasing multiple pro-inflammatory and pro-oxidative agents, and triggering a systemic inflammatory response. Moreover, adipokines secreted by visceral fat participates in the occurrence of metabolic dysfunctions.

## Liver fat accumulation

Liver, as one of the major organ of ectopic fat deposition, plays a crucial role in the IR as well. Fat accumulation in liver increases fatty acid oxidation and promotes oxidative stress, and further leads to steatosis of hepatocytes. Moreover, the accumulation of hepatic fat can directly drive metabolic dysfunction through several key mechanisms. Excessive hepatic fat can disrupt the secretion and signaling of fibroblast growth factor 21 (FGF21), as well as the alteration of bile acid synthesis and secretion. Both FGF21 and bile acids play a critical role in the regulation of lipid and glucose metabolism through interactions with specific receptors, such as the farnesoid X receptor (FXR). Dysregulation of FGF21 and bile acid signaling due to hepatic fat accumulation can impair these metabolic regulatory functions, further exacerbating IR and metabolic disturbances.

Various cytokines and hormones are released by liver as well, which exacerbates systemic inflammation and IR. IR and hyperglycemia may result in the dysregulation of lipoprotein, especially in the pancreas, where the accumulation of fatty acids further reduces insulin secretion, potentially leading to type 2 diabetes. Systemic inflammatory statues not only decrease insulin sensitivity, causing impaired glucose tolerance and then metabolic disorders, but also promotes the lasting development of endothelial dysfunction and thus related complications such as hypertension, micro- and macro-vascular diseases.^[3]^ Pro-inflammatory and pro-oxidative mediators released into the systemic circulation can accelerate the progression of CVD such as atherosclerosis and myocardial damage.^[4]^ They are closely related to micro-vascular changes in the kidney as well, further increasing the risk of hypertension and renal failure.^[5]^

## Other organic ectopic fat deposition

**Figure 1:**
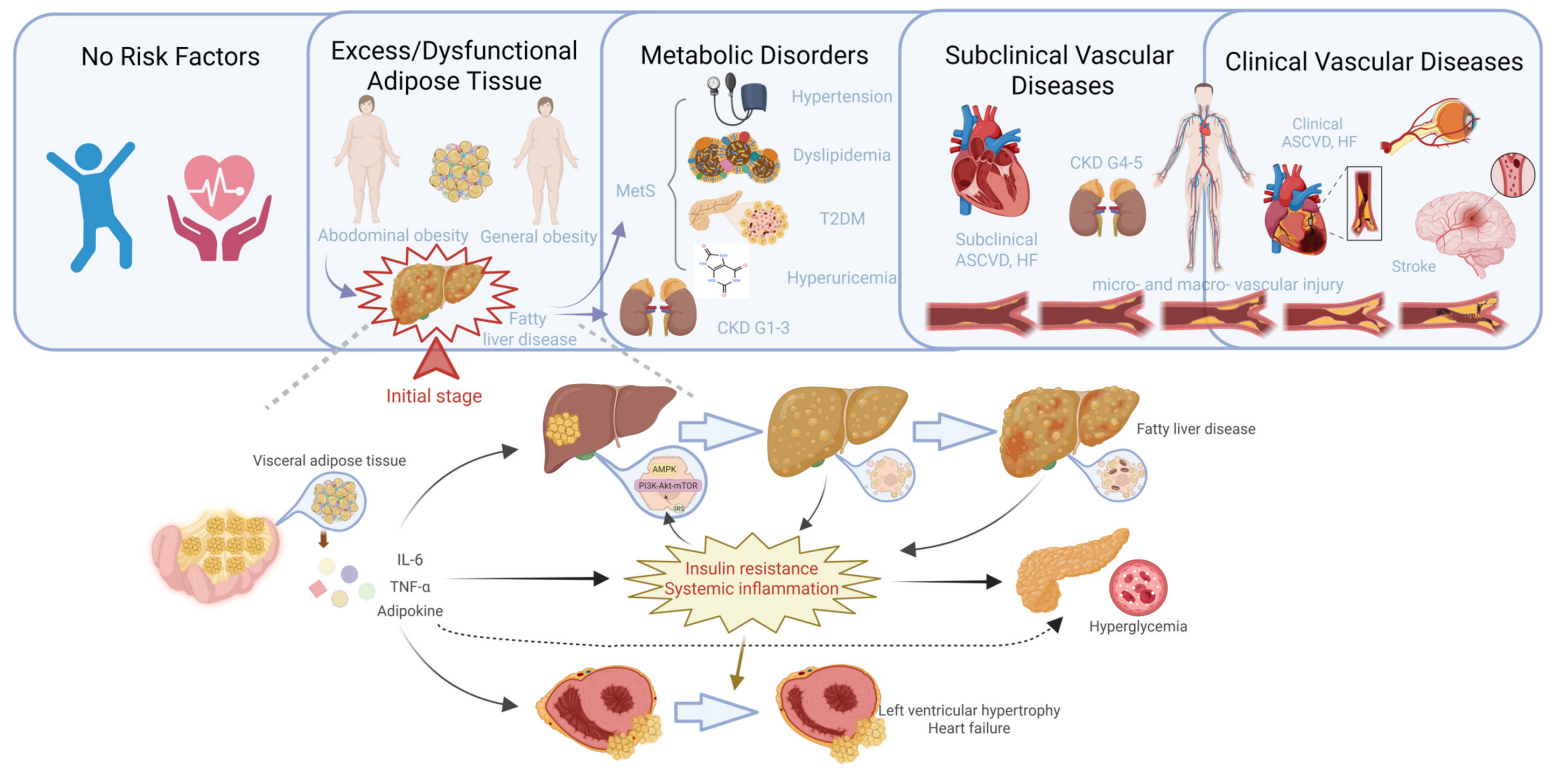
Liver adipose deposition in the framework of liver-metabolic-vascular syndrome. The deposition of adipose tissue in subcutaneous and ectopic sites leads to overall and abdominal obesity. Visceral adipose deposition further contributes to the development of fatty liver disease and metabolic disorders such as metabolic syndrome. This process sets off a vicious cycle that ultimately results in vascular dysfunction throughout the body, not just in the cardio-renal system, but also including peripheral artery disease in the limbs, intracranial vascular lesions, and cognitive dysfunction related to the central nervous system. LMV: liver-metabolic-vascular syndrome; T2DM: type 2 diabetes; CKD: chronic kidney disease; ASCVD: atherosclerotic cardiovascular disease; HF: heart failure; IL-6: interleukin-6; TNF-α: tumor necrosis factor-α.

Ectopic fat deposition in target organic also impacts the function of the heart and kidneys.^[6]^ Fat deposition within and around the kidney can directly lead to abnormal blood pressure. Epicardial and pericardial adipose tissue is associated with the incidence of cardiovascular diseases and can compress and cause heart damage. The heart’s sensitivity to visceral fat makes it more likely to left ventricular hypertrophy and heart failure under the influence of fat deposition.^[7]^ Therefore, intervention strategies for CKM should focus on the management of visceral adipose tissue (VAT) and excess ectopic fat deposition more than simply weight control, to improve overall metabolic health and reduce the risk of vascular complications (Figure1).

## Management of liver-metabolic-vascular syndrome

On the basis of CKM, we propose the concept of LMV syndrome should be acknowledged, as it introduces a paradigm shift that better reflects the natural progression of the metabolism and diseases. It identifies hepatic steatosis as the key driver of metabolic dysfunction, mediated by FGF21 resistance and bile acid dysregulation. Therefore, it underscores the need for liver-centric screening in metabolically high-risk populations, even in the absence of obesity. Metabolic disorders then progress into widespread vascular damage through complex physiological mechanisms, in contrast to CKM, which focuses primarily on downstream cardio-renal diseases. Both CKM and LMV focus on the association of metabolic disorders with organ damage. However, CKM centers on the heart and kidney functions, and LMV takes the liver as a key starting point and extends to pan-metabolic vasculopathy. LMV syndrome has potential for early intervention and etiological management, but more clinical evidence is needed to support it (See comparison in Table 1). Although the current CKM paradigm inadequately addresses hepatic contributions, its clinically validated staging system offers a robust foundation for multidisciplinary care. The proposed LMV syndrome extends this framework by introducing liver-centric biomarkers, thereby facilitating precision screening for metabolic-vascular disease progression.

**Table 1:** The extension and differences of LMV on the basis of CKM concept.

Aspects	CKM	LMV	Key Difference
Core mechanism	Heart-kidney-metabolic interactions	Liver systemic fat → vascular metabolic disease dysfunction →	LMV origin emphasizes point liver as
End point	Primarily cardio-renal diseases	pan-metabolic vasculopathy (heart, brain, peripheral arteries *etc*.)	LMV has broader coverage
Disease scope	BMI, blood glucose, kidney function	Liver fat (*e.g*., MRI-PDFF), visceral fat	LMV liver fat requires detection specific
Diagnostic focus	Staged management after metabolic abnormalities	Early liver fat screening + pre-metabolic intervention	LMV acts earlier
Intervention timing	Supported by AHA guidelines (2023)	Based validation) on NAFLD research (needs more	CKM more established

LMV: liver-metabolic-vascular; CKM: cardiovascular-kidney-metabolic; BMI: body mass index; MRI-PDFF: MRI-proton density fat fraction; AHA: American Heart Association; NAFLD: non-alcoholic fatty liver disease.

Ectopic fat depots and metabolic syndrome, as risk factors for pan-metabolic vasculopathy, undoubtedly have significant implications for the early diagnosis and treatment of target organs which can be approached through six key strategies from our perspective.

Health education should be priority. Communities and healthcare workers should take on the responsibility of disseminating risk factors of LMV, especially liver adipose deposition to the public, thereby emphasizing the importance of early detection and raising the preventive awareness. For individuals with metabolic disorder hazards, necessary examinations for target organ function and metabolism statues should be informed to help take appropriate preventive measures.

Second, timely screening and diagnosis of LMV. Life’s Essential 8, which includes eight lifestyle components, can be utilized for screening of adverse cardiovascular health.^[8]^ Early-stage adipose deposition and steatosis in liver often goes unnoticed as it is usually asymptomatic, particularly in non-obese and individuals with normal liver function. The non-invasive diagnostic methods recommended by current guidelines, such as ultrasonography, are not sensitive enough, while the costs for tests like MRI-proton density fat fraction (MRI-PDFF) are relatively high.^[9]^ Common and representative indicators should be explored, thereby developing more practical models specific to the detection of ectopic fat deposition and metabolic dysfunction.

Third, individuals with metabolic risk factors should promptly adopt lifestyle modifications to address unhealthy habits such as sedentary habits, high fat and oil diets, and alcohol consumption, which contribute to the accumulation of visceral fat. These changes should be maintained as a lifelong commitment.^[10]^

Fourth, both medical and surgical approaches to enhancing metabolism and weight loss represent a significant frontier in current and future clinical practice. When it comes to the selection of interventions, it’s important to shift the focus beyond mere weight loss to include considerations of bone density, muscle mass, and the effect of VAT. Medications or agents that do not introduce additional burdens or hazards, should be prioritized. For individuals who do not benefit from other interventions, weight loss surgery may be considered as an alternative therapeutic approach.

Fifth, LMV syndrome involves multiple organs and metabolic disturbances, requiring multidisciplinary collaboration in patient assessment and treatment planning, not only in the early stages (focused on endocrinology and gastroenterology) but also during disease progression (increased involvement of cardiology and nephrology). Additionally, a comprehensive evaluation system incorporating multidisciplinary indicators into a composite endpoint assessment can provide a holistic view of the patients’ health status and guide treatment adjustments.

Finally, LMV exhibits distinct clustering patterns within populations, necessitating community-wide management strategies beyond individual-level interventions. Targeted screening programs should be implemented in high-prevalence communities to identify at-risk individuals. Future studies are needed to evaluate the efficacy of LMV-specific interventions (*e.g*., hepatic fat reduction therapies combined with metabolic control) in improving outcomes for these populations.

## Conclusion

In summary, the CKM concept provides a vital foundation for vascular prevention, while our proposed LMV syndrome advances this framework by establishing hepatic steatosis as the pivotal trigger in metabolic-vascular pathogenesis, expanding disease scope to pan-metabolic vasculopathy (heart-brain-kidney-peripheral vessels), and as well, introducing liver-centric biomarkers for precision screening. This paradigm shift allows earlier multi-organ risk prevention *via* combined liver-metabolic-vascular interventions. Achieving this requires coordination among primary care, hepatology, and vascular specialties to improve population health.
